# Probiotic Oxalate-Degrading Bacteria: New Insight of Environmental Variables and Expression of the oxc and frc Genes on Oxalate Degradation Activity

**DOI:** 10.3390/foods11182876

**Published:** 2022-09-16

**Authors:** Dina Karamad, Kianoush Khosravi-Darani, Amin Mousavi Khaneghah, Aaron W. Miller

**Affiliations:** 1 Department of Food Technology Research, National Nutrition and Food Technology Research Institute, Shahid Beheshti University of Medical Sciences, P.O. Box 19395-4741, Tehran 1981619573, Iran; 2 Department of Fruit and Vegetable Product Technology, Professor Wacław Dąbrowski Institute of Agricultural and Food Biotechnology—State Research Institute, 36 Rakowiecka St., 02-532 Warsaw, Poland; 3 Departments of Urology and Immunology, Cleveland Clinic, Cleveland, OH 44195, USA

**Keywords:** probiotic bacteria, oxalate-degrading, variables, in vivo, in vitro, oxaluria

## Abstract

Oxalate, a compound produced by many edible plants and as a terminal metabolite in the liver of mammals, is a toxin that has a detrimental role to human health. Humans and other mammals do possess enzymatic systems to degrade oxalate. Moreover, numerous oxalate-degrading bacteria reside in the mammalian gut and, thus, provide an important function for hosts. The current review focuses on the environmental factors that influence the efficacy of probiotic oxalate-degrading bacteria, relative to oxalate metabolism. We describe the mechanism of oxalate catabolism and its consumption by obligate and facultative anaerobic oxalate-degrading bacteria, in both in vitro and in vivo environments. We also explore the environmental variables that impact oxalate degradation. Studies on single species degrade oxalate have not shown a strong impact on oxalate metabolism, especially in high oxalate conditions such as consumption of foods high in oxalate (such as coffee and chocolate for humans or halogeton in animal feed). Considering effective variables which enhance oxalate degradation could be used in application of effective probiotic as a therapeutic tool in individuals with hyperoxaluria. This study indicates probiotics can be considered a good source of naturally occurring oxalate degrading agent in human colon.

## 1. Introduction

There are several bacteria inhabiting human gut that can degrade significant amounts of oxalate daily [1]. Use of oxalate-degrading bacteria to reduce urinary oxalate has been the focus of numerous studies, with limited success [2,3]. In the human gastrointestinal tract (GIT), there are approximately four hundred different bacterial species with the composition of the gut microbiome exhibiting large, inter-individual variability [4]. Oxalate-degrading bacteria, when present in the GIT tract are able to decrease urine oxalate up to 40% and significant reduction of oxalate stone formation in the kidneys. Consumption of oxalate rich plant foods and increased digestive absorption of free oxalate can cause kidney stone formation, oxalosis, inflammation, breast cancer, atherosclerosis and cardiovascular diseases [5].

Hypercalciuria (urinary excretion of more than 800 mg of calcium per day) and hyperoxaluria (urinary excretion of more than 400 mg of oxalate per day) are among the most important pathophysiologic causes of kidney stone formation. They are directly related to calcium-oxalate rich diet. In addition, the mentioned complications result in 50% increase in calcium and oxalate concentration in urinary tracts, as well as increased level of insoluble precipitates of Ca^2+^ oxalate or phosphate in the kidney [6].

Oxalosis can leads to kidneys fail. Extra oxalate which cannot be removed from human body will accumulate in blood and different organs [3]. Prolonged exposure of breast epithelial cells to oxalate may cause tumor due to expression of proto-oncogene and increase in the proliferation rate of breast cancer cells [7].

The aim of this review was to evaluate the effective probiotic bacteria (lactic acid bacteria (LAB) and *Oxalobacter* (*O.*) *formigenes*) in the breakdown of oxalate to reduce oxalate excreted in the urine. In order to increase and improve the performance of these bacteria, effective variables, such as pH, glucose concentration, sucrose concentration, yeast extract, presence of inulin as prebiotic, bacterial age and bacterial inoculation, have been identified and studied.

### Chemistry of Oxalate

**Figure 1:**
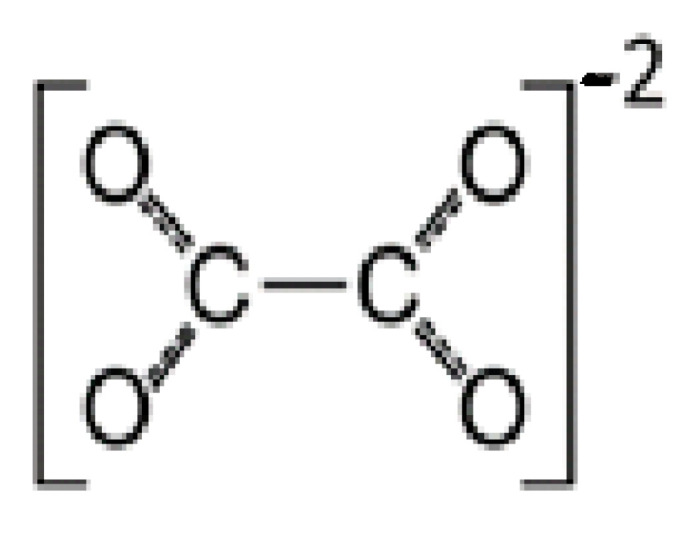
Chemical structure of oxalate anion.

Oxalate is the anion of a dicarboxylic acid that is commonly found in many plant foods, including nuts, fruits, vegetable, grains and legumes. Different salts of oxalate are found in the plants, such as sodium, potassium or magnesium oxalate, each with unique water solubility characteristics [8]. Enzymatic synthesis of oxalate occurs by hydrolysis of oxaloacetate in fungi, e.g., *Aspergillus niger*, and bacteria, e.g., *Acetobacter*. In mammals, oxalate is produced through the tricarboxylic acid cycle. The chemical structure of the anion is shown in Figure 1 [5]. A different form of oxalic acid (H_2_C_2_O_4_, HC_2_O^4−^, C_2_O_4_^2−^) may occur depending on the pH of solution. H_2_C_2_O_4_ and C_2_O_4_ are the predominant form of oxalate at pH 1.23 and 4.19 (and above), respectively.

## 2. Oxalate Sources in the Body

**Figure 2:**
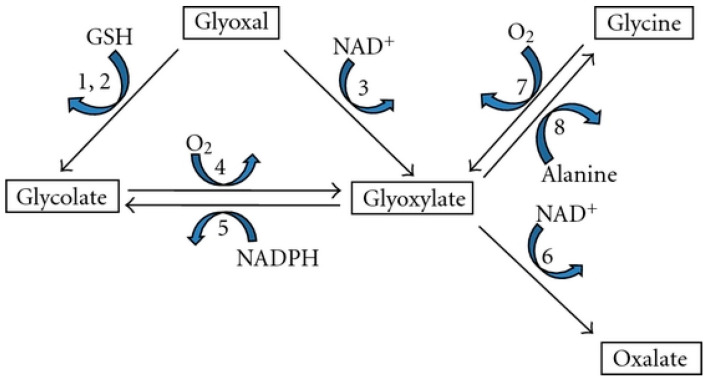
Oxalate synthesis pathway from glyoxal. In this mechanism, enzymes: alanine: glyoxylate and aminotransferase are active. (1) glyoxylase I, (2) glyoxylase II, (3) aldehyde dehydrogenase, (4) glycolate oxidase, (5) glyoxylate reductase, (6) lactate dehydrogenase, (7) D-amino acid oxidase and (8) alanine: glyoxylate aminotransferase.

The oxalate in the body has two sources: from dietary sources or from endogenous synthesis [9]. The endogenous synthesis takes place mainly within the liver, from different dietary precursors, such as glyoxalase, ascorbic acid and some amino acids [10]. Oxalate synthesis in the body has essential impact on the rate of oxalate content in the urine and formation of calcium oxalate stone in kidney. Glyoxylate is the major precursor to oxalate production. The main sources of in vivo glyoxylate metabolism are phenylalanine, glycine, hydroxyproline, tryptophan, pentose sugars, glucose, fructose, ethanolamine and glycolate [11,12,13]. Metabolism of oxalate formation from the glyoxal precursor is performed according to the cycle shown in Figure 2 [14].

All these dietary precursors are metabolized to oxalate in order to produce NADH [15]. The human body lacks any enzyme to degrade oxalate and kidneys are the main routes for eliminating oxalate from the body [16]. Recently, it has been shown that different segments of the mammalian intestine have the ability to secrete oxalate in some condition.

Short-circuited tissue preparations from rabbits, rats and mice have revealed segment-specific oxalate handling along the mammalian intestinal tract. Generally, the small intestine and proximal colon secrete oxalate under control conditions, while the distal colon absorbs oxalate. In the distal colon, oxalate can be secreted in a net amount [17].

Detoxification is carried out by the liver via two pathways—phase I and phase II. During phase I, things are broken down, then the raw materials are sent to phase II, which builds new substances by adding molecules to the raw materials (this is called conjugation). Diet needs to supply the ‘special conjugation substances’ otherwise production lines will stop. Sulfotransferase (SULT) and other phase II pathways can be negatively affected by oxalate problems. A conjugation pathway is a group of phase II pathways. In this process, fat-soluble toxic chemicals are converted into water-soluble toxins. Afterward, they are excreted in body fluids such as bile or urine [12].

## 3. Oxalate Content Estimation Methods

There have been a number of methods reported for measuring oxalate in both inoculated and noninoculated media, including titration with two titrators (potassium permanganate and NaOH) and enzymatic methods (oxalate assay kit). It was previously possible to detect acid using a known base in an acid-base reaction using the technique of titration. In addition, it can be applied to reactions involving both oxidation and reduction. Sodium oxalate in the media equals potassium permanganate in titration with potassium permanganate. Before inoculation, the media does not undergo biodegradation, resulting in a higher volume of permanganate for the balance titration point. This method only allows us to estimate the sodium oxalate content, which is equivalent to potassium permanganate, which is the limitation of this method. Due to the additional methods and calculations required for oxalate determination, this method would be time-consuming and expensive [18].

The Oxalate Assay kit is a colorimetric method with a high level of sensitivity, ease of use and adaptability. This assay involves reacting oxalate with an intermediate, which can then be analyzed by spectrophotometry. Oxalate levels between 0.05 and 0.7 mmol can be detected using the assay kit. Due to the faster analysis time, enzymatic methods are preferred over titration methods for estimating the oxalate content of foods with a medium (0.05 mmolL^−1^) to high (100 mmol L^−1^) oxalate content. Analyzing dietary oxalate content accurately will provide information about dietary oxalate’s role in urinary oxalate excretion and stone formation [19].

## 4. Degradation of Oxalate by Obligate and Facultative Anaerobic Gut Bacteria

There is considerable inter-individual variability in the composition of the gut microbiota, but generally remains stable within individuals. Gut microbiota composition can, however, differ over time in individuals with varying diets and other factors, such as antibiotic use. Oxalate-degrading bacteria in the gut are able to decrease oxalate (as a source of carbon and energy) by 40% and reduce oxalate stone formation in kidney. The absence of oxalate degrading bacteria in the GIT had shown to be a risk factor for the hyperoxaluria and urolithiasis [16,20].

**Figure 3:**
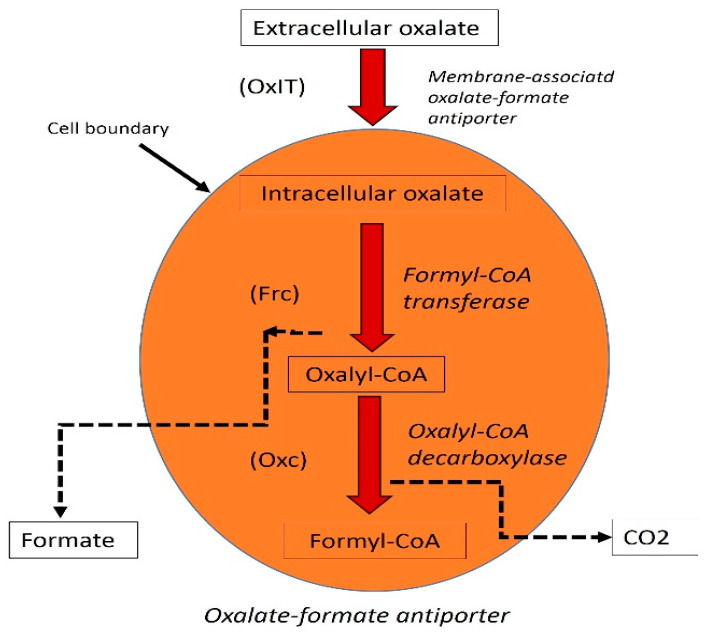
Oxalate degradation pathway in *O. formigenes*. Enzyme names are italicized with their protein abbreviations in brackets; dotted lines show secreted product.

It has been isolated for the first time by Alison et al. It consumes oxalate as a source of carbon and energy and is found in the human digestive system and other vertebrates [21]. *O. formigenes* highly susceptible to common antibiotics. Normally, the reproduction rate of *O. formigenes* is higher in people without kidney stones than in people with kidney stones. There is no clear relationship between *O. formagenes* proliferation and urinary oxalate secretion [22,23,24]. There is a possibility that patients with primary hyperoxaluria will not respond to the probiotic *O. formigenes* [2]. It has been shown that individual *O. formigenes* were lost after therapeutic use of antibiotics and other drugs as well as in patient with cystic fibrosis [1,25]. *O. formigenes* with anaerobic oxalate degrading activity can degrade oxalic acid with three enzyme (Figure 3) [23].

Furthermore, different investigations showed that probiotic bacteria, especially *Bifidobacterium* (*B.*) spp. and *Lactobacillus* (*L.*) spp. (Table 1), have the ability of degradation oxalate into carbon dioxide and formate. *Lactobacillus* and *Bifidobacteria* are Gram-positive, non-spores and rod-shaped species and are found in large numbers in the human intestine. *Bifidobacteria* are anaerobic, while *Lactobacillus* species are often highly tolerant to air. Since some strains of these bacteria (as mentioned in Table 1) are in the safe group for human consumption, different species of these two bacteria are widely used as probiotic bacteria to improve human health [1]. It should be noted that the breakdown of oxalate in *Lactobacilli* and *Bifidobacteria* is specific to the genus and species of certain bacteria. In 2022, Jiang et al. [26] studied the decomposition of oxalate in a wide range of human microbiota especially *Bifidobacterium* spp. They found that oxalate oxidase, decarboxylase, frc, and oxc are the key oxalate-degrading enzymes. Oxalate decarboxylases and oxalate oxidases are members of the cupin superfamily of proteins and the two enzymes show high similarity at the amino acid level.

## 5. Variables Affecting the Activity of Oxalate Degrading Bacteria

### 5.1. pH

Azcarate-Peril et al. [30] reported transcription of genes (oxc and frc) in *L. acidophilus* which was stimulated at pH 5.5 and inhibited at pH 6.8 in the presence or absence of oxalate. Lewanica and colleagues showed that *L. gasseri* at pH 5.5 can decompose 74% of oxalate. Moreover, in the simulated colon medium, Lactobacillus reduces the fraction of 40% of oxalate in the culture medium (at 60 h and pH 5.5) [31]. Karamad et al. [32] showed that in *L. acidophilus* at pH 5.5 and with increasing sodium oxalate concentration from 5 mmolL^−1^ to 22.7 mmolL^−1^ sodium oxalate decomposition shows an increase. At this pH, the bacterium has the highest expression of the oxalate degradation gene and due to the pre-adaptation of the bacterium to high oxalate content the oxalate degradation ability of bacterium significantly increased [32]. They also showed that in *O. formigenes* ammonium oxalate degradation rate increases with increasing pH from 5.5 to 6. In pH less than 6 oxalate degradation drops sharply [33].

### 5.2. Glucose and Sucrose Concentration

Tarruni et al. [14] showed that *Bifidobacteria* were unable to grow and degrade oxalate, and found this by completely recovering oxalate from the growth medium after 5 days in the incubator (37 °C). Growth retardation has also occurred due to the lack of oxalate and sucrose in the bacterial growth medium [14]. They also showed that rapidly proliferating cells consumed carbon sources (30% decomposition, 0.25 gL^−1^h^−1^) during the first 24 h. After 24 h until 120 h of incubation, after logarithmic growth, the residual sucrose is slowly reduced to half the initial concentration (0.07 gL^−1^h^−1^). In in vivo mouse study, Miller et al. [34] showed that that both antibiotics and high fat, high sugar diet lowers microbial oxalate metabolism.

### 5.3. Yeast Extract

Dawson et al. [35] showed that removing yeast extract from the growth media reduced the relative growth rate of *O. formigenes* by 80% in 4 days. In this experiment, it was finally shown that the only energy source needed (other than oxalate) for the growth of *O. formigenes* is yeast extract, and the best amount of yeast added to the growth medium is 0.1%. So that if this rate increases to 0.2%, it will not have an effect on increasing bacterial growth.

### 5.4. Bacterial Age

In a study, Gholami and Khosravi Darani [36] showed that the age of inoculation is 36 h and the inoculation rate of 0.8% leads to higher production of dual linoleic acid.

### 5.5. Inulin

Previous research by Balthazar et al. [37] has shown that among prebiotics, inulin may cause increase survival and activity of LAB during shelf life. Stepanova et al. [38] showed that impact of prebiotics (oligofructose and inulin) on increased degradation of oxalate is due to the increased growth of bacteria resulted from the presence of short chain free fatty acids. Another study by Darilmaz et al. [39] showed that prebiotics could increase the degradation of oxalate by lactobacilli in vitro. Inulin also plays a key role in the anti-*E. coli* activity, which can be increased by the use of *Limosilactobacillus fermentum* IP5. Karamad et al. [32] found from their research on *L. acidophilus* that by increasing the amount of inulin from 0.5 gL^−1^ to 0.97 gL^−1^ at pH: 5.5, the highest rate of oxalate decomposition has been performed (about 90% of 20 mmolL^−1^ sodium oxalate concentration). They also showed that in *O. formigenes* as the concentration of inulin increases, the rate of oxalate degradation increases and its optimal value was 1.35 gL^−1^. In fact, this study for the first time investigated the effect of inulin on the oxalate degradation activity of *O. formigenes* [33].

### 5.6. Antibiotics

There is a strong correlation between antibiotic therapy and kidney stones disease and oxalate degrading bacteria population in colon (Table 2). Although specific mechanisms have not been identified, it is clear that *O. formigenes* is antibiotic sensitive. Absence of intestinal *O. formigenes* could represent a pathogenic factor in calcium oxalate urolithiasis when antibiotics are prescribed generously [40,41,42].

## 6. Analysis of Transcription and Function of the oxc and frc Genes in LAB and *O. formigenes*

The genes for oxalyl-CoA decarboxylase (oxc) and formyl-CoA transferase (frc) play a key role in oxalate metabolism in *O. formigenes* and LAB were isolated by Allison [21]. By utilizing oxc and frc as catalysts in a two-step enzymatic reaction, oxalate can be metabolized into CO_2_ and formate.

pH and oxalate exposure may interact directly to affect oxalate degradation, but may also have wider effects on microbial community dynamics and function. In *L. acidophilus*, 315 genes are down-regulated with exposure to 1% oxalate at pH 6.8, and 16 genes are up-regulated with exposure to 1% oxalate at pH 6.8 [28]. Under these conditions, oxc and frc, which degrade oxalate, are down-regulated. The flow of oxalate between gut regions with varying pH can affect gene expression in whole microbial communities using next-generation metagenomic strategies. Using this technique, oxalate-induced shifts in microbiota function and community composition could be predicted more accurately. Several efforts have been made in this field, including the sequencing of *O. formigenes* as part of the human microbiome project (Broad Institute). Oxalate degradation is particularly sensitive to pH, and the cyclic fatty acid configuration of *O. formigenes* indicates a degree of acid tolerance in this species [43].

The oxalyl-CoA decarboxylase function was attributed to the product of the open reading frame (ORF) based on amino acid similarity with proteins of known function. The oxalyl-CoA decarboxylase of *O. formigenes* (accession no. M77128) presented the highest nucleotide homology (56%) and amino acid similarity (identities, 47%; positives, 64%). Furthermore, most of the decarboxylase enzymes described to date, including the oxalyl-CoA decarboxylase of *O. formigenes*, present a conserved thiamine pyrophosphate (TPP)-binding region [26].

**Figure 4:**
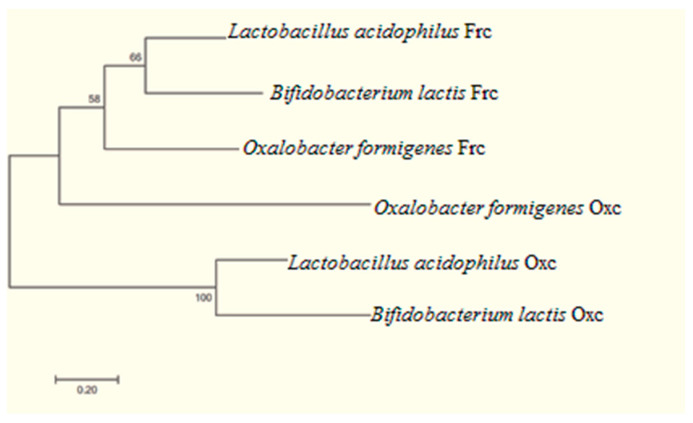
Molecular phylogenetic analysis by maximum likelihood method.

The current understanding of the phylogenetic relatedness of *O. formigenes* with *L. acidophilus* and *B. lactis* is summarized in Figure 4.

The evolutionary history was inferred by using the Maximum Likelihood method based on the Tamura–Nei model [44]. The tree with the highest log likelihood (−4566.2828) is shown. The percentage of trees in which the associated taxa clustered together is shown next to the branches. Initial tree(s) for the heuristic search were obtained automatically by applying Neighbor-Join and BioNJ algorithms to a matrix of pairwise distances estimated using the Maximum Composite Likelihood (MCL) approach, and then selecting the topology with superior log likelihood value. The tree is drawn to scale, with branch lengths measured in the number of substitutions per site. The analysis involved six nucleotide sequences. Codon positions included were 1st + 2nd + 3rd + Noncoding. All positions containing gaps and missing data were eliminated. There were a total of 634 positions in the final dataset. Evolutionary analyses were conducted in MEGA7 [45]. In this analysis, we use bootstrap method and the number of bootstrap applications is 1000 [46].

## 7. Discussion

Consumption of probiotic bacteria can be a suitable treatment method for people with kidney stone disease and individuals with high risk of infection. *L. acidophilus* and *O. formigenes* have shown good results [1,16]. However, more research is needed on the appropriate amount and conditions of use of these dietary supplements to achieve the highest rate of oxalate degradation, especially when consuming high oxalate foods. In previous studies [33,34], it has been shown that the simultaneous use of variables affecting the decomposition of oxalate and can greatly increase oxalate degradation by probiotic bacteria in high oxalate content.

In the postnatal period, colonic anaerobes play an important role in the development and functioning of the organism. If we are able to recognize the functions performed by colonic bacteria, we should be able to develop therapies under medical supervision that can be administered to individuals lacking key bacteria in the future. The degradation of toxic compounds in the intestine provides ecological niches for anaerobic bacteria in the gut [35]. Both the human colon and the animal gut are thought to offer an ecological niche for oxalate degradation. The potential for replacement therapy with probiotic preparations of *O. formigenes* should be excellent in such a niche [47]. In these investigations, molecular quantitative methods can be used since Oxalobacter’s loss correlates with other diseases, in addition to the potential link between antibiotic use and its loss [48] will be helpful. Other than LAB and *O. formigenes*, it seems unclear what bacteria are responsible for oxalate degradation in the gut [9,27]. In studying microbial oxalate degradation in the intestinal tract, one of the main goals is to reduce the incidence of recurrent renal colic, an economically damaging condition.

There are four key enzymes that degrade oxalate: oxidase, decarboxylase, frc and oxc. The oxalate decarboxylase and oxalate oxidase belong to the cupin superfamily of proteins, which show strong similarities at the amino acid level. There were significantly more genes encoding frc and oxc in the gut than genes encoding oxalate oxidase and decarboxylase. According to the analysis of 660 subjects, the four genes encoding the enzymes were widely present in the healthy gut microbiome [49]. In the metagenomes of 660 subjects, oxc can be detected in 554 (84%) and frc in 581 (88%). According to Jiang et al. [26], oxc can be found in a multitude of bacterial genomes and metagenomes, providing information about its presence, classification, and phylogenesis. Furthermore, they analyzed the enzyme’s abundance throughout the human microbiome, which is not limited to the gut. A final step was the purification and characterization of two enzymes. In bacteria and in human microbiomes, oxcs are widely distributed. In the human body, bacteria with oxcs are found in different ecological niches, even though oxcs have been highly conserved throughout evolution.

Gut microbiota play an important role in gut-kidney physio-pathology. Inhibiting urinary stone disease by maintaining healthy oxalate homeostasis could be achieved by a multi-species bacterial network [34]. In addition to research on isolated species of oxalate-degrading bacteria, particularly those that require oxalate to function, recent studies indicate that microbiota play broader roles in oxalate metabolism and in inhibiting urinary stone formation. It has been shown that the highest biodegradation by *O. formigenes* DSM 4420 was achieved in presence of 1.48 (gL^−1^) inulin, 44.82 (gL^−1^) glucose, 16.04 (mmol L^−1^) ammonium oxalate and pH 6.5. Reconfirmation experiment showed the validity of predicted optimum conditions [33]. They also showed that ideal condition for *L. acidophilus* ATCC 4356 to degrade oxalate are included inulin (0.987 g L^−1^), sodium oxalate (22.796 m L^−1^), glucose (37.46 g l^−1^) and pH (5.5) [33]. Consumption of enough bacteria seems to be an efficient tool for prevention of formation of oxalate kidney stone in high risk individuals.

## 8. Conclusions

Consumption of probiotic bacteria can be a suitable treatment method for people with kidney stone disease and individuals with a high risk of infection. A review of all reports showed that *L. acidophilus* and *O. formigenes* have shown promising results in these studies. However, more research is needed on the appropriate amount and conditions of using these dietary supplements to achieve the highest rate of oxalate degradation, especially when consuming high oxalate foods. Studies indicate that the simultaneous use of variables affecting the decomposition of oxalate can significantly increase oxalate degradation by probiotic bacteria in high oxalate content.

## Figures and Tables

**Table 1:** Oxalate degrading bacteria in *Bifidobacterium* and *Lactobacillus* sp.

Microorganisms	Sources	Reference
*Lactiplantibacillus plantarum*	YOMO Research Centre	[27]
*Lactobacillus brevis*		
*Lactobacillus acidophilus*		
*Bifidobacterium infantis*		
*Bifidobacterium animalis* ATCC 27536		
*Bifidobacterium breve* MB 283	Gut intestine human	[28]
*Bifidobacterium longum* MB 282		
*Bifidobacterium infantis* MB 57		
*Bifidobacterium adolescentis* MB 238		
*Lacticaseibacillus casei*	Gut intestine human	[29]
*Lactobacillus acidophilus*	Gut intestine human	[30]
*Lactobacillus gasseri* Gasser AM63T	Gut intestine human	[31]
*Lactobacillus acidophilus*		
*Lactobacillus gasseri*		
*Lactiplantibacillus plantarum*		
*Lacticaseibacillus casei*		
*Lacticaseibacillus rhamnosus*		
*Ligiactobacillus salivarius*		

**Table 2:** Antibiotic sensitivity of *O. formigenese* strains (R = resistant; S = sensitive).

Antibiotic	HC1	Va3	Cc13	OxK
Amoxicillin	R	R	R	R
Amoxicillin/Clavulanate	R	R	R	R
Azithromycin	S	S	S	S
Ceftriaxone	R	R	R	R
Cephalexin	R	R	R	R
Ciprofloxacin	S	S	S	S
Clarithromycin	S	S	S	S
Clindamycin	S	S	S	S
Doxycycline	S	S	S	S
Gentamicin	S	S	S	S
Levofloxacin	S	S	S	S

## Data Availability

Not applicable.
